# Tumor-derived exosomal tsRNA 3′tiRNA-AlaCGC in promoting fibroblast senescence and Galectin-9 secretion to induce immune tolerance in lung adenocarcinoma

**DOI:** 10.1038/s41420-025-02695-3

**Published:** 2025-08-25

**Authors:** Guangyin Zhao, Yuchen Zhang, Hongyu Zhang, Yifan Guo, Chang Xu, Di Ge, Jie Gu

**Affiliations:** 1 Department of Thoracic Surgery, Shanghai Geriatric Medical Center, 2560 Chunshen Road, Shanghai, China; 2 Department of Thoracic Surgery, Zhongshan Hospital, Fudan University, Shanghai, China

**Keywords:** Tumour immunology, Senescence

## Abstract

Given the heterogeneity of the tumor microenvironment (TME), neoadjuvant immunotherapy combined with chemotherapy benefits only a subset of lung adenocarcinoma (LUAD) patients, and the mechanisms of resistance remain unclear. Transfer RNA-derived small RNAs (tsRNAs) are a new class of non-coding RNAs that participate in the remodeling of the TME. Using high-throughput small RNA microarray analysis, we found elevated expression of tsRNA 3′tiRNA-AlaCGC in tumors of LUAD patients resistant to neoadjuvant therapy, and negatively correlated with the poor prognosis in LUAD patients. Furthermore, we discovered that tumor-derived exosome carrying 3′tiRNA-AlaCGC target fibroblasts to induce a senescence-associated secretory phenotype (SASP) by inhibiting FOXO3, and activating the TGF-β/Smad3 pathway, thereby increasing Galectin-9 secretion; both SASP and Galectin-9 induce synthetically dysfunction of cytotoxic CD8^+^ T cells. In vivo experiments revealed that high expression of 3′tiRNA-AlaCGC led to decrease infiltration and diminished cytotoxic function of CD8^+^ T cells in tumors of C57BL/6 mice, resulting in anti-PD-L1 therapy resistance. Collectively, our research underscores the immunosuppressive role of 3′tiRNA-AlaCGC in LUAD, offering insights into its molecular traits and aiding personalized treatment strategy development.

## Introduction

The advent of immunotherapy has significantly transformed the treatment landscape for driver gene-negative LUAD patients [1]. Immune checkpoint inhibitors (ICIs) such as anti-PD-1 or PD-L1 monoclonal antibodies have demonstrated notable efficacy in advanced-stage LUAD patients, propelling immunotherapy into the realm of neoadjuvant therapy [2–4]. However, a minority of LUAD patients exhibit significant responses to immunotherapy, impacting prognosis. Investigating the molecular mechanisms of immunotherapy resistance, exploring effective therapeutic targets, enhancing efficacy, and improving the prognosis of LUAD patients remain urgent.

Under stress conditions, tRNA can be cleaved by endonucleases, generating fragments known as tRNA-derived small RNAs (tsRNAs) [5, 6]. Historically, tsRNAs have been regarded as random degradation products without functional significance [7]. However, with advancements in sequencing technologies, research on the functions of tsRNAs has gradually deepened, confirming their important roles in both physiological and pathological processes, including cancer [8–11]. In breast cancer, a class of endogenous tRNA fragments has been found to displace the 3’UTR of target mRNA from the RNA-binding protein YBX1, thereby inhibiting the stability of multiple oncogenic transcripts in breast cancer cells. These fragments share a common motif that matches the YBX1 recognition sequence. Loss-of-function and gain-of-function studies have revealed that these fragments can inhibit the proliferation, invasion, and metastasis of breast cancer cells [12]. In a mouse liver cancer model, the tRNA fragment LeuCAG3′tsRNA promotes the translation of ribosomal protein mRNAs (RPS28 and RPS15) by binding to them, and inhibition of this tsRNA induces tumor cell apoptosis [10]. These findings indicate that tsRNAs are not randomly generated non-functional fragments. They can influence the stability and translation of mRNA in tumors, exhibiting a dual role in both inhibiting and promoting cancer progression. However, research on tsRNAs is still in its early stage.

Cellular senescence is a complex biological process within organisms, involving a decline in cell functions leading to a cessation of cell division [13]. Although senescent cells stop dividing, they are not entirely inactive; they affect their surrounding cells and tissue environment by secreting various bioactive molecules, a phenomenon known as the senescence-associated secretory phenotype (SASP) [14, 15]. An excessive accumulation of senescent cells or an imbalance in their secreted SASP molecules can promote inflammatory responses, tissue damage, and even the development of cancer [16]. Within the tumor microenvironment (TME), the role of SASP molecules is particularly complex [16, 17]. On one hand, they can inhibit tumor growth by inducing inflammatory responses and attracting immune cells [18]. On the other hand, certain SASP molecules might facilitate tumor cell proliferation, invasion, and metastasis, helping tumors evade immune surveillance and even promoting angiogenesis [15]. Therefore, understanding the mechanisms and effects of SASP can provide crucial insights for developing new anti-cancer strategies [19].

Here, high-throughput small RNA microarray detection revealed an upregulation of tsRNA 3′tiRNA-AlaCGC in resistant LUAD tumors under neoadjuvant therapy (NCT04316364), with its high expression associated with poor prognosis in LUAD patients. We discovered that tumor-derived exosome carrying 3′tiRNA-AlaCGC target fibroblasts, inducing a senescence-associated secretory phenotype (SASP) and activating the TGF-β/Smad3 pathway to increase Galectin-9 secretion; both SASP and Galectin-9 are known to induce CD8^+^T cell exhaustion. This study aims to elucidate the specific mechanisms by which exosomal 3′tiRNA-AlaCGC reshapes the TME and induces resistance to immunotherapy in LUAD, using clinical samples and in vivo and in vitro models, providing a rationale for tsRNA as a predictive marker for immunotherapy efficacy and a therapeutic target in LUAD.

## Results

### High 3’tiRNA-AlaCGC expression in LUAD correlates with poor immunotherapy response and prognosis

**Fig. 1:**
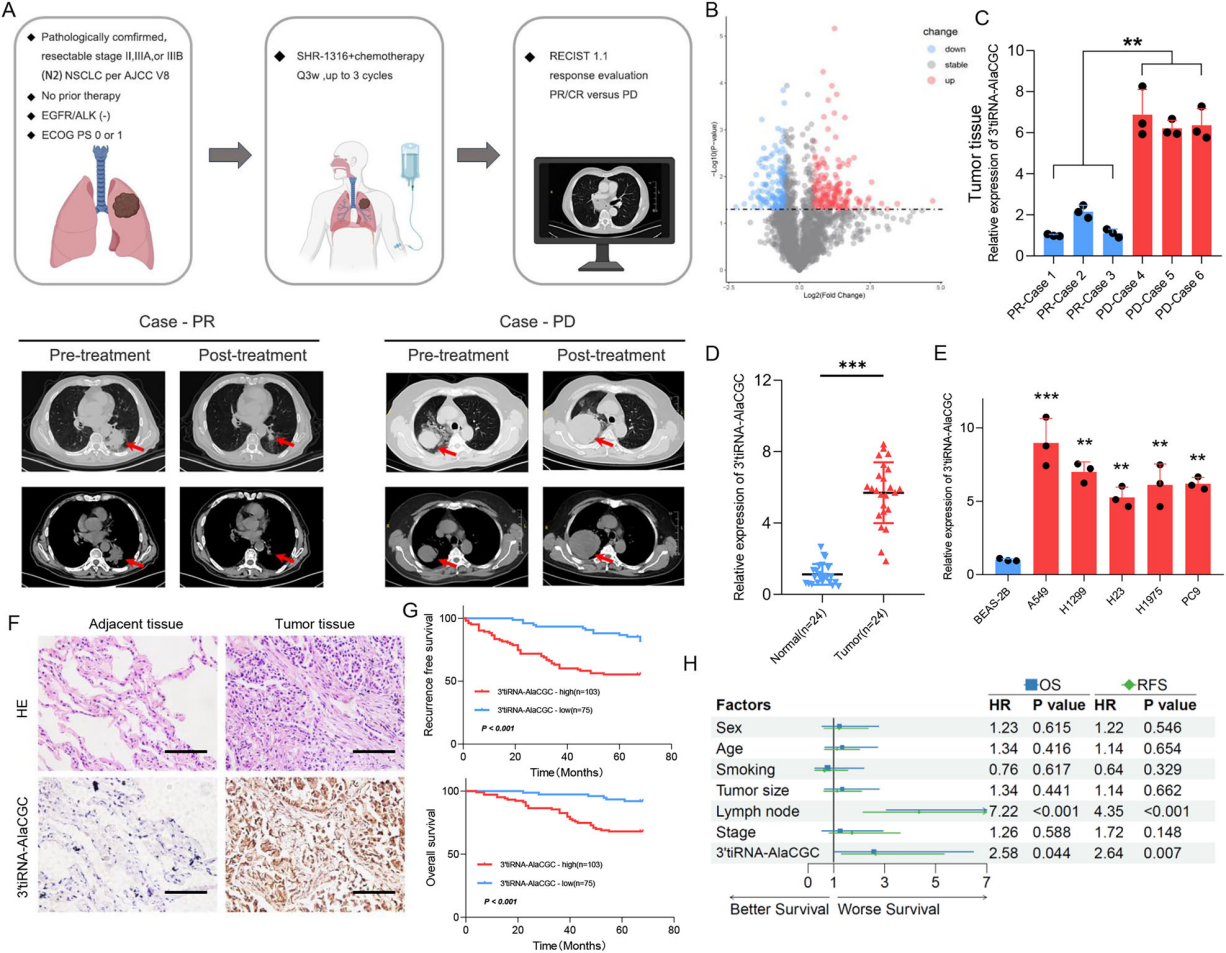
High 3′tiRNA-AlaCGC expression in LUAD correlates with poor immunotherapy response and prognosis. **A** Schematic diagram of neoadjuvant therapy in LUAD Clinical Trials (Upper Section) and comparative imaging of treatment responses: Partial Response (PR) versus Progressive Disease (PD) (Lower Section). **B** Volcano plot illustrating differential expression of tsRNAs in tumor tissues with varying treatment responses. **C** qPCR validation of tsRNA 3′tiRNA-AlaCGC expression differences in tumor tissues of LUAD patients between PR and PD groups. **D** Differential expression of 3′tiRNA-AlaCGC in tumor tissues and adjacent non-tumor tissues from 24 pairs of fresh postoperative LUAD cases. **E** Differential expression of 3′tiRNA-AlaCGC between tumor cell lines and normal bronchial epithelial cell lines. **F** The expression of 3′tiRNA-AlaCGC between adjacent normal tissues and tumor tissues from patients with LUAD. **G**, **H** Tissue microarray analysis revealed that LUAD patients with high 3′tiRNA-AlaCGC expression have poorer RFS and OS. Each experiment was performed three times independently and results are presented as mean ± s.d. Student’s *t*-test was used to analyze the data. (*p < 0.05; **p < 0.01; ***p < 0.001).

Based on the clinical trial evaluating SHR-1316, a PD-L1 monoclonal antibody, or placebo combined with chemotherapy for preoperative treatment of resectable Stage II-III non-small cell lung cancer (a randomized, double-blind, multi-center Phase IB/III trial, NCT04316364), Arraystar Small RNA microarray (Aksomics, Shanghai) analysis was performed on pre-treatment LUAD samples from 3 cases of progressive disease (PD) and 3 cases of partial response (PR), according to RECIST v1.1 criteria (Fig. 1A). Differentially expressed tsRNAs were shown in Fig. 1B, with the top 10 most significantly altered tsRNAs further validated by qPCR. Notably, 3’tiRNA-AlaCGC demonstrated both significant differential expression and stability (Supplementary Fig. 1A, B). We then selected the top 3 tsRNAs with the fold change greater than 10 for validation in tumor and adjacent non-tumor tissues from LUAD patients. The results revealed that 3’tiRNA-AlaCGC was significantly upregulated in tumor tissues and exhibited the most pronounced differential expression (Supplementary Fig. 1C, D, and E). Consequently, 3’tiRNA-AlaCGC was selected for further experimental investigation.

Further qPCR analysis confirmed that, compared to the PR group, 3’tiRNA-AlaCGC was upregulated in both tumor tissues and plasma of PD patients (Fig. 1C and Supplementary Fig. 1F).

Correlation analysis revealed a good match between 3’tiRNA-AlaCGC qPCR and microarray results (Supplementary Fig. 1G). We next collected 24 pairs of LUAD tumors and adjacent normal lung tissues. qPCR analysis also showed that 3’tiRNA-AlaCGC expression was significantly higher in LUAD tumor tissues than in normal lung tissues (Fig. 1D). Similarly, the expression of 3’tiRNA-AlaCGC was significantly higher in NSCLC cell lines than that in normal bronchial epithelial cells (Fig. 1E). We further analyzed the relationship between 3’tiRNA-AlaCGC expression and the prognosis of NSCLC patients by in situ hybridization using tissue microarrays (TMA) (Fig. 1F). The clinicopathological characteristics of this cohort of patients were presented in Table 1. The results revealed that high expression of 3’tiRNA-AlaCGC was associated with significantly shorter DFS and OS in LUAD patients than in patients with low expression (Fig. 1G). Cox regression indicated that 3’tiRNA-AlaCGC expression was an independent prognostic factor in LUAD patients (Fig. 1H).

**Table 1:** Clinicopathologic variables in 178 patients with LUAD.

Variable	No. of patients	3′tiRNA-AlaCGC expression		*P*
		Low	High	
Age				
≤61	98	45	53	0.258
>61	80	30	50	
Sex				
Male	71	28	43	0.553
Female	107	47	60	
Smoking status				
Yes	26	9	17	0.401
No	152	66	86	
Stage				
I-II	153	71	82	0.004
III-IV	25	4	21	
Lymph node metastasis				
Yes	46	9	37	<0.001
No	132	66	66	
Tumor size				
≤3 cm	133	68	65	<0.001
>3 cm	45	7	38	

### The 3’tiRNA-AlaCGC expression does not affect LUAD cell proliferation, migration, and invasion at the cellular level

**Fig. 2:**
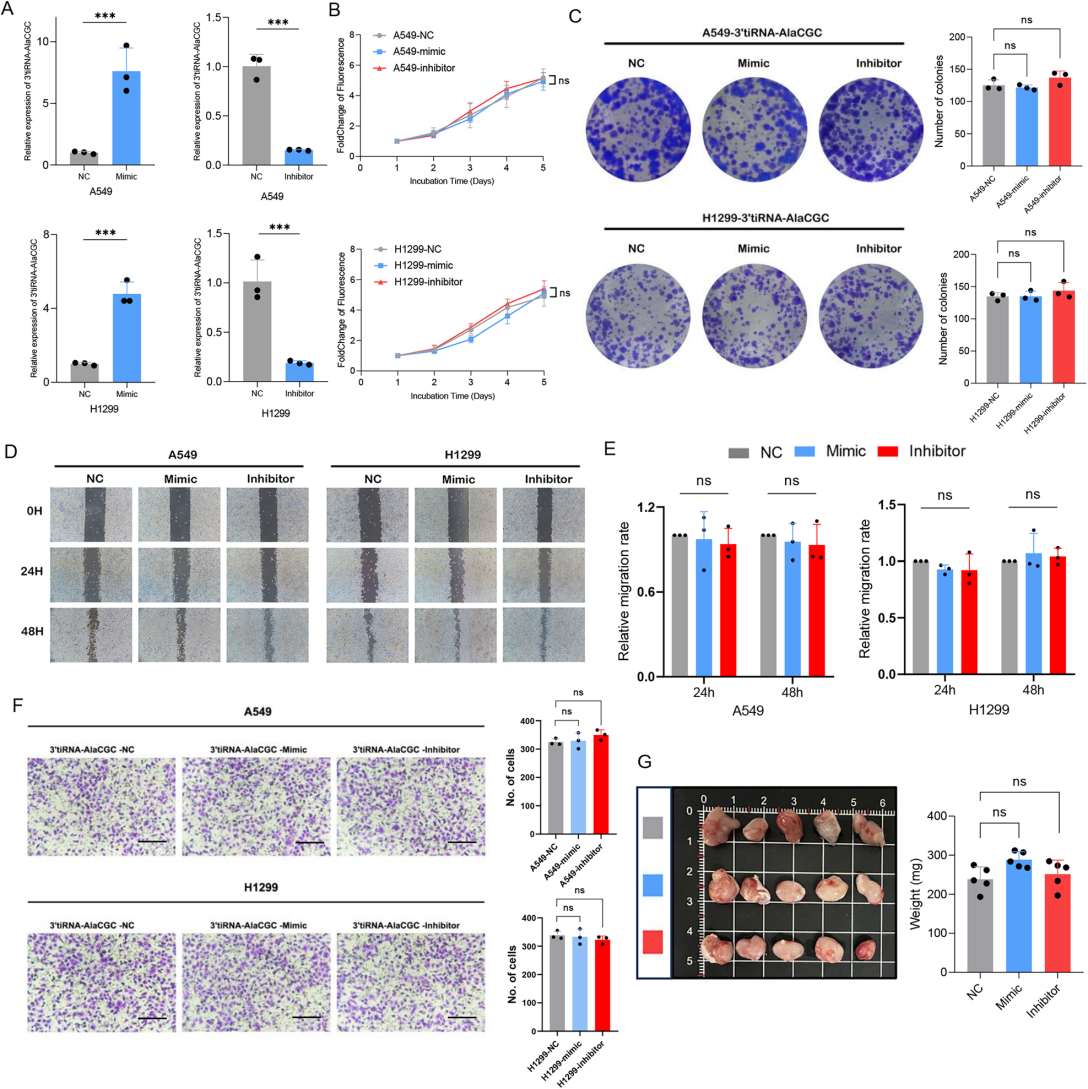
The 3′tiRNA-AlaCGC expression has no affection in LUAD cell proliferation, migration, or invasion at the cellular level. **A** q-PCR confirming the overexpression and knock-down of 3′tiRNA-AlaCGC in A549 and H1299 cells. **B**, **C** The CCK-8 and colony formation assays confirmed the impact of dysregulation of 3′ tiRNA-AlaCGC expression on the proliferation ability of A549 and H1299 cells. **D**, **E** The wound healing assay verified the impact of changes in 3′tiRNA-AlaCGC expression on the migration ability of A549 and H1299 cells. **F** The Transwell assay verified the impact of 3′ tiRNA-AlaCGC on the invasive capabilities of A549 and H1299 cells. **G** Representative tumors formed in nude mice by A549-3′tiRNA-AlaCGC-NC, A549-3′tiRNA-AlaCGC-mimic, A549-3′tiRNA-AlaCGC-inhibitor cells. Each experiment was performed three times independently and results are presented as mean ± s.d. Student’s *t*-test was used to analyze the data. (*p < 0.05; **p < 0.01; ***p < 0.001).

Given that 3’tiRNA-AlaCGC is associated with poor response to immunotherapy and unfavorable prognosis in LUAD patients, to determine whether it influences the biological behavior of LUAD, we further validated this through cellular experiments. Firstly, we transfected LUAD cell lines (A549 and H1299) with the mimic or inhibitor (The sequence was provided by Genomeditech, Shanghai, Supplementary Table 3), which altered the intracellular level of 3’tiRNA-AlaCGC. Transfection efficiency was detected by qPCR (Fig. 2A). Then, we performed CCK-8 assays and plate clone formation assays to explore the effects of 3’tiRNA-AlaCGC on LUAD cell proliferation. Our results demonstrated that different expression levels of 3’tiRNA-AlaCGC had no significant effect on the proliferative capacity of both A549 and H1299 cells (Fig. 2B, C). In addition, the 3’tiRNA-AlaCGC expression did not affect the migratory as well as invasion ability of A549 and H1299 cells (Fig. 2E-F).

To validate the impact of 3’tiRNA-AlaCGC on LUAD in vivo, different A549 cells (3’tiRNA-AlaCGC-NC, 3’tiRNA-AlaCGC-mimic, 3’tiRNA-AlaCGC-Inhibitor) were injected subcutaneously to create subcutaneous tumor formation in nude mice. Consistent with the above results, there were also no significant differences in tumor size among the three groups of nude mice (Fig. 2G). Consequently, our data suggest that 3’tiRNA-AlaCGC does not influence the proliferative, migratory, and invasive capabilities of LUAD cells in vitro, nor does it affect tumorigenesis under conditions of immunodeficiency in vivo.

### Tumor-derived 3’tiRNA-AlaCGC induces senescence in fibroblasts via the exosomal pathway

**Fig. 3:**
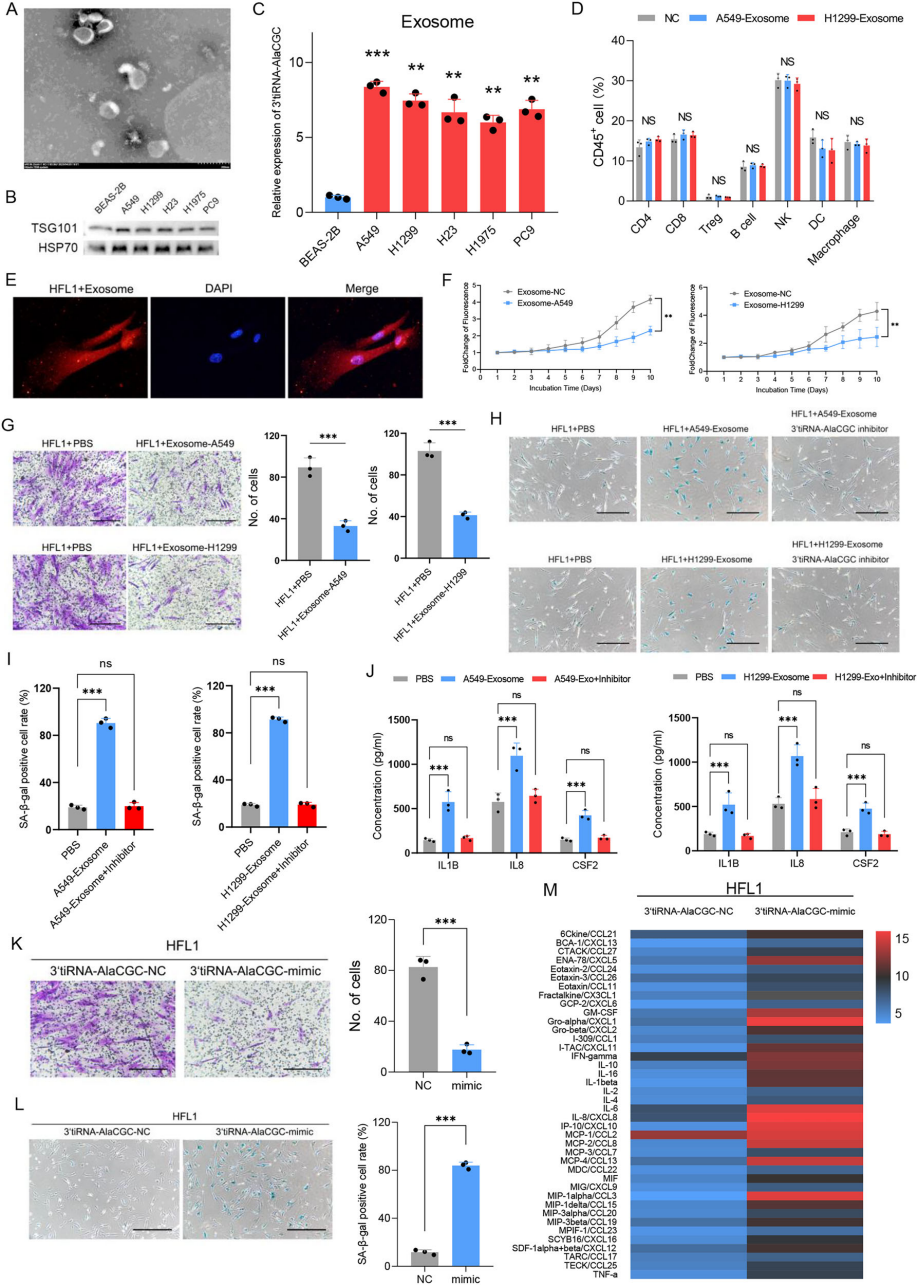
The 3′tiRNA-AlaCGC induces senescence in fibroblasts via the exosomal pathway. **A**, **B** Exosomes released by different cancer cells were detected by electron microscopy and western blotting analysis. **C** qPCR analysis of 3′tiRNA-AlaCGC expression in exosomes released by different cells. **D** Sorting of CD45^+^ immune cells from the periphery of healthy donors and analyzing changes in various immune cell components after treatment with tumor-derived exosomes. **E** Fluorescence microscopy displayed a typical image of fibroblasts HFL1 ingesting tumor-derived exosomes (A549 and H1299). **F** Changes in the proliferation capacity of HFL1 cells after treatment with exosomes derived from A549 and H1299 cells. **G** Changes in the migration ability of HLF1 cells after treatment with exosomes derived from A549 and H1299 cells. **H**, **I** SA-β-gal staining assessed senescence in HFL1 cells after treatment with exosomes derived from A549 and H1299 cells and 3′tiRNA-AlaCGC inhibitors. **J** The status of typical SASP factors secreted by HFL1 cells after treatment with exosomes derived from A549 and H1299 cells and 3′tiRNA-AlaCGC inhibitors. **K** Changes in the migration ability of HFL1 cells with overexpression of tsRNA via mimic transfection. **L** SA-β-gal staining assessed senescence in HFL1 cells after overexpression of 3′tiRNA-AlaCGC. **M** Liquid-phase cytokine array analysis between HFL1-NC and HFL1-mimic. Each experiment was performed three times independently and results are presented as mean ± s.d. Student’s *t*-test was used to analyze the data. (*p < 0.05; **p < 0.01; ***p < 0.001).

Cell-cell communication plays a pivotal role in the tumor microenvironment (TME), influencing various aspects of cancer progression, metastasis, and response to therapy [20, 21]. Exosomes represent a crucial pathway for intercellular interactions within the TME [21]. Therefore, we hypothesize that tumor cell-derived 3’tiRNA-AlaCGC may influence surrounding immune cells via the exosomal pathway, leading to poor responses to immunotherapy. We isolated exosomes secreted by LUAD and normal bronchial epithelial cells and successfully verified them through electron microscopy and western blot (Fig. 3A, B). In line with our hypothesis, we detected the levels of 3’tiRNA-AlaCGC in exosomes derived from tumor cells, which was significantly higher than that in exosomes produced by normal epithelial cells (Fig. 3C). Then, we collected peripheral blood from three healthy donors, isolated CD45^+^ cells, co-cultured them with LUAD exosomes for 48 h, and examined the changes in the proportions of the individual immune cell components by flow cytometry assay. Intriguingly, the proportion of individual immune cells in exosome-treated CD45^+^ cells was not significantly different compared to the control group (Fig. 3D). We further speculated whether 3’tiRNA-AlaCGC exerts its effects on stromal cells, focusing particularly on fibroblasts, the main component of stromal cells within the TME, which subsequently became the subject of our experiments. We cultured human lung fibroblasts HFL1, and after co-culturing them with tumor-derived exosomes, we found that HFL1 cells could uptake exosomes (Fig. 3E), and the proliferative and migration capacity of HFL1 was significantly inhibited after treatment with A549-derived or H1299-derived exosomes (Fig. 3F, G). Furthermore, exosome-treated fibroblasts have a higher proportion of senescent state (Fig. 3H, I). Thus, we have concluded preliminarily that LUAD-derived exosomes can cause HFL1 cells to undergo senescence. SASP is a major marker of senescence, Senescence β-Galactosidase Staining demonstrated that the intake of tumor-derived exosomes significantly increased the senescence ratio in HFL1 cells, along with a significant increase in major senescence-associated cytokines. However, this process was reversed upon the addition of a 3′tiRNA-AlaCGC inhibitor (Fig. 3H-J). By upregulating the level of 3′tiRNA-AlaCGC in HFL1 cells through synthetic mimic, a decrease in HFL1 cell migration ability and an increase in senescence was observed (Fig. 3K, L). Liquid-phase cytokine array analysis between HFL1-NC and HFL1-mimic revealed that 3′tiRNA-AlaCGC leads to a transformation of HFL1 towards an SASP (Fig. 3M).

### Exosomal 3′tiRNA-AlaCGC promotes senescence in fibroblasts by down-regulating the expression of FOXO3

**Fig. 4:**
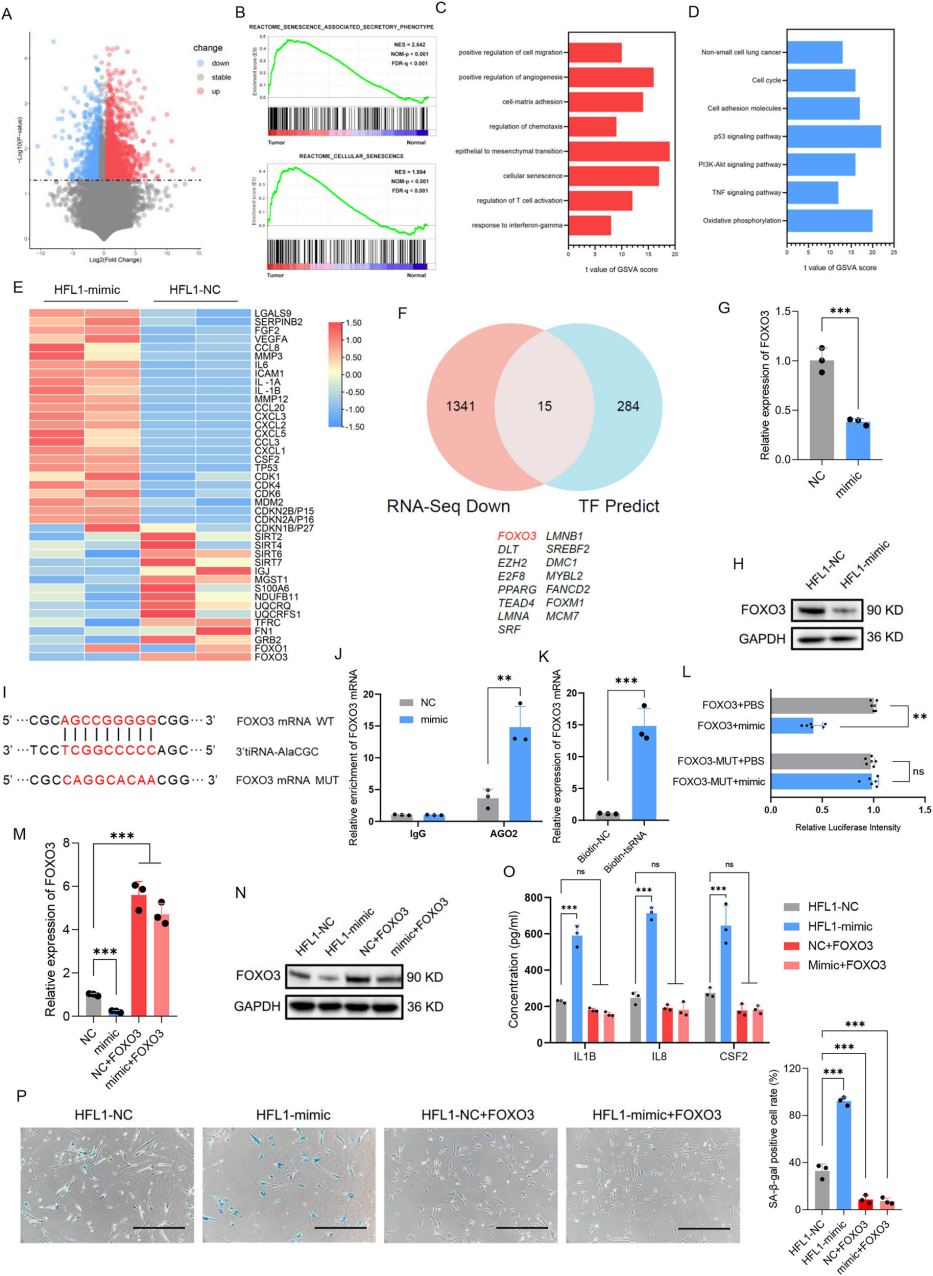
The 3′tiRNA-AlaCGC promotes senescence in fibroblasts by down-regulating the expression of FOXO3. **A** High-throughput RNA-Seq on HFL1-NC and HFL1-mimic cells, with differential gene expression displayed in a volcano plot. **B** GSEA graphs for the upregulation of SASP phenotype and senescence-associated genes in HLF1 cells after overexpression of 3′tiRNA-AlaCGC. **C**, **D** GO and KEGG pathway analyses also showed significant enrichment of pathways related to cell senescence. **E** Heatmap displayed the expression changes of SASP-related genes and major longevity gene sets in HFL1 cells after overexpression of 3′tiRNA-AlaCGC. **F** A dataset of transcription factors associated with SASP was compiled and intersected with our RNA-seq differential gene dataset. **G**, **H** qPCR and western blotting assessed differences in FOXO3 gene expression in HFL1 cells after overexpression of 3′tiRNA-AlaCGC. **I** Sequence alignment of 3′tiRNA-AlaCGC and FOXO3 mRNA using SeqBuilder revealed potential base-pairing complementarity between tsRNA and the CDS region of FOXO3. **J**, **K** RIP and RNA pull-down assays were used to determine the interaction between 3′tiRNA-AlaCGC and FOXO3 mRNA. **L** Dual luciferase activity assays to analyze the differences in fluorescence intensity before and after mutations in the potential binding region of FOXO3 with 3′tiRNA-AlaCGC, following treatment with 3′tiRNA-AlaCGC mimic. **M**, **N** qPCR and western blotting validated the efficiency of restoring FOXO3 expression. **O** After restoring FOXO3 expression, the typical SASP factors secreted by HFL1 cells promoted by 3′tiRNA-AlaCGC were antagonized. **P** SA-β-gal staining assessed the senescence in HFL1 cells after restoring FOXO3 expression. Each experiment was performed three times independently and results are presented as mean ± s.d. Student’s *t*-test was used to analyze the data. (*p < 0.05; **p < 0.01; ***p < 0.001).

To elucidate the mechanism by which 3′tiRNA-AlaCGC, once uptaken by HFL1 cells, induces senescence, we performed high-throughput RNA-Seq on HFL1-NC and HFL1-mimic cells. Differentially expressed genes are presented in Fig. 4A. GSEA enrichment analysis revealed significant enrichment of the senescence phenotype, while GO and KEGG pathway analyses also showed significant enrichment of pathways related to senescence (Fig. 4B-D). Furthermore, upon upregulation of 3′tiRNA-AlaCGC expression, a significant increase in the expression of genes related to the SASP in HFL1 cells was noted (Fig. 4E). We identified transcription factors associated with SASP and compiled a corresponding dataset. By intersecting this dataset with our RNA-seq differential gene dataset, a notable downregulation of FOXO3, known as a longevity-associated gene [22], was observed in HFL1-mimic cells (Fig. 4F). qPCR and western blot experiments also validated the negative regulatory relationship of 3′tiRNA-AlaCGC with FOXO3 (Fig. 4G, H). Since the tsRNAs can act through base pairing with complementary sequences on target mRNAs, leading to the inhibition of translation, similar to the action of microRNAs (miRNAs), or it can lead to the degradation of the target mRNA [9, 11, 23]. Consequently, sequence alignment revealed that the core sequence of 3′tiRNA-AlaCGC shows strong base complementarity with the coding sequence (CDS) region of FOXO3 mRNA (Fig. 4I), Further RIP and RNA pull-down experiments subsequently confirmed that tsRNA directly binds to FOXO3 mRNA (Fig. 4J, K). By mutating the sequences within the mRNA CDS region that bind to 3′tiRNA-AlaCGC and employing a Dual-Luciferase reporter assay, we confirmed that 3′tiRNA-AlaCGC can down-regulate FOXO3 by binding to the CDS region of mRNA (Fig. 4I, L). Subsequently, we restored FOXO3 expression (Fig. 4M, N) and confirmed through ELISA and Senescence β-Galactosidase Staining that overexpression of FOXO3 in HFL1 cells significantly inhibited the senescence phenotype induced by 3′tiRNA-AlaCGC (Fig. 4O, P). Thus, we concluded that LUAD cells induce senescence in fibroblasts by secreting exosomal 3′tiRNA-AlaCGC that targets and binds to FOXO3 mRNA, suppressing its expression and leading to a senescent phenotype transformation in the fibroblasts.

### Tumor-derived exosomal 3′tiRNA-AlaCGC enhances Galectin-9 expression through activation of the TGF-β-SMAD3 pathway

**Fig. 5:**
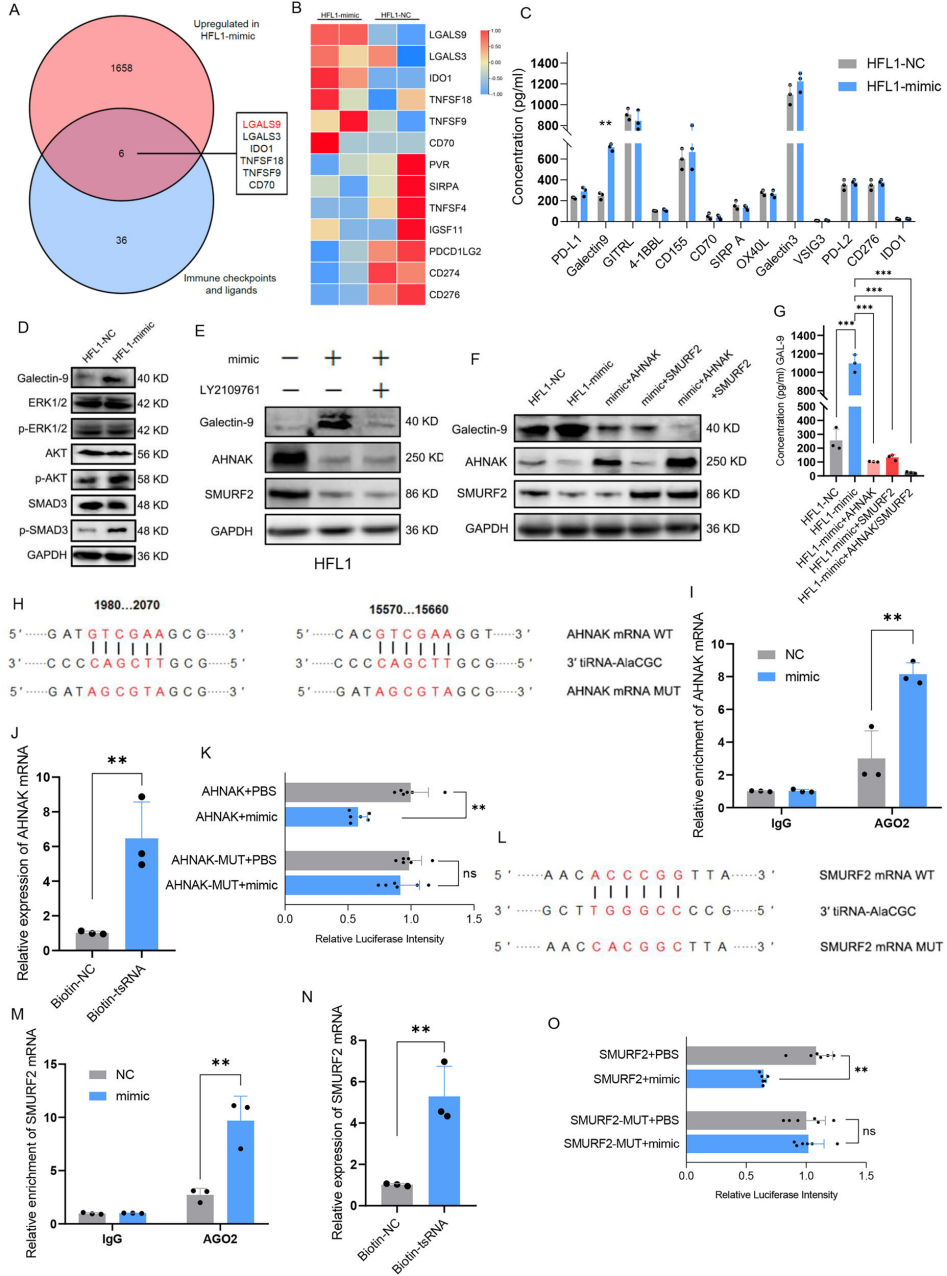
The 3′tiRNA-AlaCGC promotes Galectin-9 expression by activating the TGF-β-SMAD3 signaling. **A** Venn plot displayed the intersection between genes upregulated in HFL1-mimic and immune checkpoint and ligand gene sets. **B** Heatmap showed the expression differences of immune checkpoint ligands between HFL1-NC and HFL1-mimic. **C** ELISA validation of the differences in levels of immune checkpoint ligands secreted by HFL1-NC and HFL1-mimic. **D** Western blotting analysis showed the phosphorylation levels of ERK1/2, AKT, and Smad3 in HFL1 cells when 3′tiRNA-AlaCGC is overexpressed. **E** Overexpression of 3′tiRNA-AlaCGC in HFL1 cells, combined with the addition of the TGF-β pathway inhibitor LY2109761$ (10 μM, 2 h), resulted in changes in the expression of Galectin-9, AHNAK, and SMURF2. **F**, **G** Significant suppression of Galectin-9 expression and secretion occurred only when both AHNAK and SMURF2 expressions were simultaneously restored. **H** Sequence alignment using SeqBuilder reveals potential base-pairing complementarity between 3′tiRNA-AlaCGC and the CDS region of AHNAK. **I**, **J** RIP and RNA pull-down assays were used to determine the interaction between 3′tiRNA-AlaCGC and AHNAK mRNA. **K** Dual luciferase activity assays to analyze the differences in fluorescence intensity before and after mutations in the potential binding region of AHNAK with 3′tiRNA-AlaCGC, following treatment with 3′tiRNA-AlaCGC mimic. **L** Sequence alignment using SeqBuilder reveals potential base-pairing complementarity between 3′tiRNA-AlaCGC and the CDS region of SMURF2. **M**, **N** RIP and RNA pull-down assays were used to determine the interaction between 3′tiRNA-AlaCGC and SMURF2 mRNA. **O** Dual luciferase activity assays to analyze the differences in fluorescence intensity before and after mutations in the potential binding region of SMURF2 with 3′tiRNA-AlaCGC, following treatment with 3′tiRNA-AlaCGC mimic. Each experiment was performed three times independently and results are presented as mean ± s.d. Student’s *t*-test was used to analyze the data. (*p < 0.05; **p < 0.01; ***p < 0.001).

Now it’s established that cancer cells can stimulate checkpoint proteins that inhibit immune responses to evade immune detection and destruction. Our study revealed a significant upregulation of the immune checkpoint ligand Galectin-9 concurrent with 3′tiRNA-AlaCGC-mediated senescence in HFL1 cells (Fig. 4E). To validate this observation, we intersected the up-regulated gene set in HFL1-mimic cells with the immune checkpoint and their ligands gene set, revealing that 3′tiRNA-AlaCGC could also potentially promote the expression of Galectin-9 in fibroblasts (Fig. 5A, B). Furthermore, ELISA assays of the principal immune checkpoint ligands showed that Galectin-9 secretion by HFL1-mimic cells was significantly higher than in the NC group (Fig. 5C). Western blot similarly verified that 3′tiRNA-AlaCGC promotes Galectin-9 protein expression in HFL1 cells (Fig. 5D). Previous research indicated that the expression of Galectin-9 is regulated by the TGF-β-SMAD3 pathway [24]. To verify whether 3′tiRNA-AlaCGC are involved in the regulation of this pathway, we examined several common pathways and found that in HFL1 cells, the up-regulation of 3′tiRNA-AlaCGC expression significantly increases the level of p-SMAD3 (Fig. 5D). Upon overexpression of 3′tiRNA-AlaCGC, the addition of the LY2109761 (10 μM, 2 h), a TGF-β-SMAD2/3 inhibitor, to HFL1 cells, inhibiting the activation of this pathway, also resulted in a decrease in Galectin-9 level (Fig. 5E). These findings indicate that 3′tiRNA-AlaCGC promotes Galectin-9 expression by activating the TGF-β-SMAD3 pathway. We also found that overexpression of 3′tiRNA-AlaCGC significantly inhibited the expression of AHNAK and SMURF2 (Fig. 5E), both of which have been confirmed as inhibitory molecules of SMAD3, capable of suppressing the activity of the TGF-β pathway [25, 26]. Significant suppression of Galectin-9 expression was observed only when the expression of both AHNAK and SMURF2 were simultaneously restored (Fig. 5F, G). Similar to the regulation of FOXO3, sequence alignment analysis revealed that 3′tiRNA-AlaCGC exhibits base complementarity with the CDS regions of both AHNAK and SMURF2 mRNAs (Fig. 5H, L). Moreover, RIP and RNA pull-down experiments also confirmed that tsRNA directly binds to AHNAK (Fig. I and J) and SMURF2 mRNAs (Fig. 5M, N). Dual-Luciferase reporter assays have also confirmed functional binding between 3′tiRNA-AlaCGC and the wild-type (WT) mRNAs of these two molecules (Fig. 5K, O). To conclude, 3′tiRNA-AlaCGC elevates Galectin-9 expression by inhibiting AHNAK and SMURF2, thereby activating the TGF-β/SMAD3 pathway.

### Tumor-derived exosomal 3′tiRNA-AlaCGC concurrently regulates the expression of FOXO3 and Galectin-9 in fibroblasts, thereby inhibiting CD8^+^T cell function

**Fig. 6:**
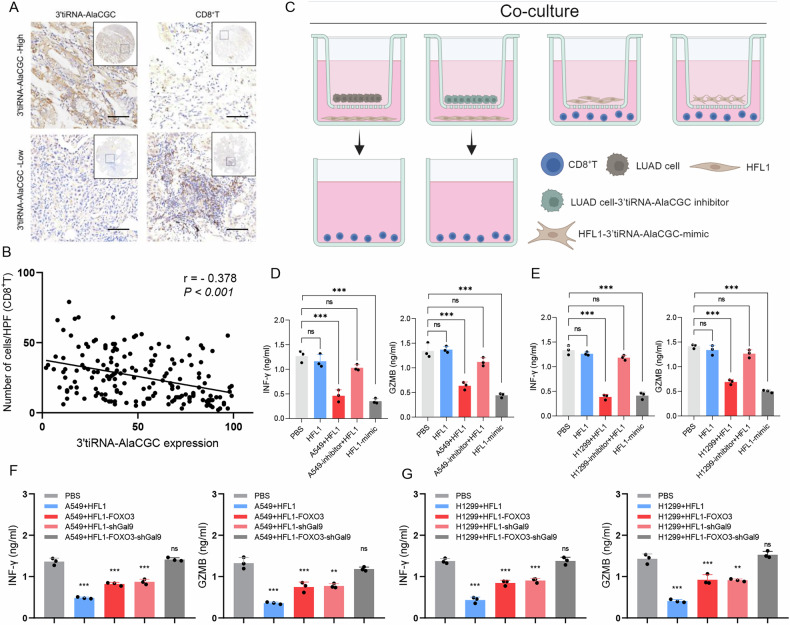
Tumor-derived exosomal 3′tiRNA-AlaCGC concurrently regulates the expression of FOXO3 and Galectin-9 in fibroblasts, thereby inhibiting CD8^+^T cell function. **A** Infiltration of CD8^+^T cells in the tissue microarray (TMA) of LUAD patients was analyzed by immunohistochemistry (IHC); scale bar, 100 μm. **B** A Spearman correlation analysis was applied to analyze the relationship between the infiltration of CD8^+^T cells and the expression of 3′tiRNA-AlaCGC. **C** Co-culture workflow for CD8^+^T cells, HFL1 cells, and LUAD cells. **D**, **E** After co-culturing CD8^+^T cells with different cells (HFL1 cells, LUAD + HFL1 cells, LUAD-3′tiRNA-AlaCGC inhibitor + HFL1 cells, HFL1-mimic cells), ELISA was used to analyze the status of its cytotoxic function. **F**, **G** By restoring or interfering with the expression of FOXO3 and Galectin-9 in HFL1 cells, followed by co-culturing with CD8^+^T cells, the changes in the cytotoxic function of CD8^+^T cells were analyzed. Each experiment was performed three times independently and results are presented as mean ± s.d. Student’s *t*-test was used to analyze the data. (*p < 0.05; **p < 0.01; ***p < 0.001).

To explore whether 3′tiRNA-AlaCGC affects immunoreactivity through fibroblasts, we next used tissue microarrays for FISH and immunohistochemical staining, analysis revealed that the expression of 3′tiRNA-AlaCGC in LUAD tissues was inversely correlated with the proportion of CD8^+^ T cells (r = −0.378, *p* < 0.001; Fig. 6A, B). Then, we conducted a co-culture system to validate that tsRNA indirectly affects CD8^+^ T cell function by regulating fibroblasts (Fig. 6C). Consistent with previous results, co-culturing CD8^+^ T cells with HFL1 cell lines does not affect their secretory function. However, when CD8^+^ T cells are treated with the supernatant from co-cultures of LUAD and HFL1 cell lines, their ability to secrete INF-γ and GZMB was significantly inhibited (Fig. 6D, E). Suppressing the 3′tiRNA-AlaCGC of LUAD cells, and co-cultured with HFL1 had no significant effect on the function of CD8^+^ T cells. However, CD8^+^ T cells co-cultured with HFL1 expressing 3′tiRNA-AlaCGC exhibited significant functional suppression (Fig. 6D, E). This indicates that LUAD inhibits CD8^+^ T cell function by targeting fibroblasts with exosomal 3′tiRNA-AlaCGC. Consequently, we further discovered that only by simultaneously restoring FOXO3 and inhibiting Galectin-9 expression in HFL1-mimic cells, the function of CD8^+^ T cells is almost completely restored under co-culture conditions (Fig. 6F, G). Therefore, LUAD cells inhibit FOXO3 and promote Galectin-9 expression and secretion in HFL1 cells via the exosomal 3′tiRNA-AlaCGC pathway, synergistically suppressing the CD8^+^ T cell function of secreting INF-γ and GZMB.

### 3′tiRNA-AlaCGC inhibits the cytotoxicity of CD8^+^ T cells and sensitizes anti-PD-L1 immunotherapy in vivo

**Fig. 7:**
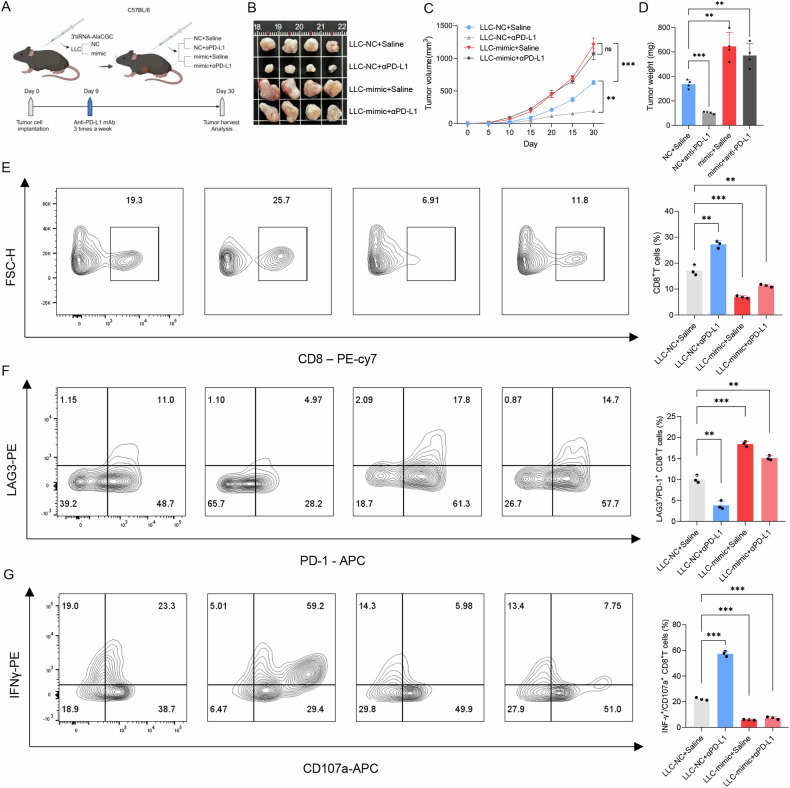
The 3′tiRNA-AlaCGC inhibits the cytotoxicity of CD8 + T cells and sensitized anti-PD-L1 immunotherapy in vivo. **A** In vivo experimental workflow. LLC tumor cells overexpressing tsRNA (mimic) and control cells (NC) were subcutaneously implanted into C57BL/6 mice, followed by treatment with or without anti-PD-L1 antibodies (αPD-L1). **B** Tumors harvested from mice bearing LLC-NC tumor cells, LLC-mimic tumor cells, LLC-NC tumor cells treated with anti-PD-L1 antibody, and LLC-mimic tumor cells treated with anti-PD-L1 antibody (*n* = 4). **C** Tumor growth curves (*n* = 4). Statistical analysis was performed using two-way ANOVA with multiple comparisons. **D** The weight of the final tumor formed between different groups. **E** Flow cytometry analysis of the proportion of CD8^+^T cells infiltrated in the tumors. **F**, **G** Flow cytometry analysis of immune effector molecules, PD-1, LAG3, CD107a, and IFNγ in CD8^+^T cells. Data were presented as mean ± s.d. Two-way ANOVA with multiple comparisons was used to analyze the data C, Student’s *t*-test was used to analyze the data **D**-**G**. (*p < 0.05; **p < 0.01; ***p < 0.001).

Given the significant impact of 3′tiRNA-AlaCGC upregulation in tumor cells on CD8^+^ T cell functionality, we further investigated whether 3′tiRNA-AlaCGC expression in tumor cells could impair CD8^+^ T cell cytotoxicity and immunotherapy responsiveness in vivo (Fig. 7A). Results showed that in immune-competent mice, tumor cells with 3′tiRNA-AlaCGC overexpression exhibited significantly enhanced proliferation compared to the control group (Fig. 7B-D). Following anti-PD-L1 treatment, significant tumor shrinkage was observed in the LLC-NC group, while the mimic group showed minimal reduction (Fig. 7B-D). Hence, it can be inferred that tsRNA presence in tumor cells contributes to immunotherapy resistance.

Subsequent flow cytometry analysis revealed that overexpression of 3′tiRNA-AlaCGC in tumor cells led to a decreased proportion of tumor-infiltrating CD8^+^ T cells. Additionally, upon anti-PD-L1 immunotherapy, the NC group exhibited a significant increase in CD8^+^ T cell infiltration within tumors, unlike the mimic group (Fig. 7E). Functional assessments of these CD8^+^ T cells demonstrated that 3′tiRNA-AlaCGC overexpression significantly inhibited their cytotoxic capabilities (Fig. 7F, G).

## Discussion

The advent of immunotherapy has profoundly transformed the treatment landscape for patients with driver gene-negative LUAD. The efficacy of immune checkpoint blockers (ICB), such as anti-PD-1 or PD-L1 monoclonal antibodies, in advanced LUAD patients, has propelled immunotherapy into the neoadjuvant treatment phase. However, only a minority of LUAD patients exhibit a significant response to immunotherapy, impacting prognosis. Addressing immunotherapy resistance has become a current research focus, presenting a challenging and lengthy endeavor. Our study was based on a randomized, double-blind, multicenter Phase IB/III trial evaluating SHR-1316 (anti-PD-L1) with platinum chemotherapy versus placebo with platinum chemotherapy in resectable Stage II/III non-small cell lung cancer, focusing on efficacy and safety. We collected pre-treatment tumor tissues from enrolled LUAD patients, analyzing the correlation between novel non-coding small RNA, tsRNA, and immunotherapy resistance, aiming to explore new mechanisms behind lung adenocarcinoma’s resistance to immunotherapy.

tRNA is one of the most abundant non-coding RNAs within cells. Under stress conditions, tRNAs can be cleaved by endonucleases, producing fragments known as tRNA-derived small RNAs (tsRNAs) [27]. Several investigations have presented evidence that tsRNAs play crucial roles in both physiological and pathological processes, including cancer [8–10]. The aberrant expression of tsRNAs in cancer cells has been linked to tumor growth, metastasis, and clinicopathological traits, and tsRNAs may serve as targets for cancer treatment in addition to being diagnostic and prognostic markers [23, 28, 29]. Similar to miRNAs, tsRNAs play a role in gene silencing, even competing with miRNAs for binding to their targets [12]. tsRNAs are also present in bodily fluids and participate in intercellular communication via exosomes. In hepatocellular carcinoma patients, tsRNA levels in plasma exosomes are significantly higher than in healthy individuals, suggesting tsRNAs’ potential as cancer diagnostic biomarkers [30]. However, whether tsRNAs are involved in the immunoregulation of tumors remains uncertain. Moreover, advancements in sequencing technology provide us with the opportunity to further explore the biological functions of this novel class of non-coding small RNAs. In the present study, we discovered the differential expression of tsRNA 3′tiRNA-AlaCGC between immunotherapy-sensitive (PR) and resistant (PD) LUAD patient tumor tissues. Notably, 3′tiRNA-AlaCGC expression was significantly higher in immunotherapy-resistant tissues, and high 3′tiRNA-AlaCGC expression in LUAD tissue microarrays was associated with poorer patient prognosis. More importantly, altering 3′tiRNA-AlaCGC levels in LUAD cells did not affect the tumor cell proliferation, migration, or invasion capabilities. Chiou et al. found that activated T cells selectively secrete tsRNAs via extracellular vesicles (EVs), which can inhibit T cell activation, thereby regulating the immune response. And, in hepatocellular carcinoma patients, levels of four tsRNAs (tRNA-ValTAC-3, tRNA-GlyTCC-5, tRNA-ValAAC-5, and tRNA-GluCTC-5) were significantly elevated in plasma exosomes, suggesting that tsRNAs can function through the exosomal pathway. We indeed observed exosomes secreted by LUAD cells express 3′tiRNA-AlaCGC, with levels significantly higher than those in exosomes from normal bronchial epithelial cells. More importantly, exosomes derived from LUAD can target fibroblasts, leading to a significant decrease in fibroblast proliferation and migration capabilities. Further investigation revealed that tumor-derived 3′tiRNA-AlaCGC induces fibroblast senescence by suppressing the expression of FOXO3.

Aging has been added as a new member to the third edition of the Hallmarks of Cancer in 2022 [31]. Its essence is cellular senescence, which refers to the cessation of cell division and the entry into a stable, non-proliferative state while remaining metabolically active [32]. This natural anti-cancer mechanism prevents damaged or aging cells from proliferating uncontrollably, maintaining homeostasis. However, senescent cells can secrete a range of bioactive molecules, such as cytokines, growth factors, and proteases, collectively known as the SASP [14]. Studies have shown that in the TME, senescent stromal cells establish a chronic inflammatory milieu through SASP, protecting tumor cells, evading immune surveillance, and leading to tumor invasion and metastasis [33, 34]. Human genetic association studies have shown that FOXO3 is linked to human longevity, termed a “longevity gene.” FOXO3 is currently the only transcription factor known to be associated with lifespan in organisms ranging from yeast, hydra, Caenorhabditis elegans, Drosophila, and mice, to humans [22, 35]. Its genetic variations are key factors affecting human aging and related diseases. Our further investigation revealed that tumor-derived 3′tiRNA-AlaCGC induces fibroblast senescence by suppressing the expression of FOXO3, restoring FOXO3 expression in HFL1-mimic cells can reverse the senescence phenotype. Moreover, 3′tiRNA-AlaCGC induces an increase in SASP secretion by fibroblasts through the suppression of FOXO3 expression, which contributes to the inhibition of CD8^+^ T cell function. This is the first study to date that has identified tumor-derived exosomal tsRNA-inducing fibroblast senescence leading to immunosuppression. Whether this pathway also plays other roles affecting the TME requires further study to elucidate.

The mechanisms underlying resistance to immune therapy are highly complex, among which the expression levels and patterns of immune checkpoint molecules can directly impact the efficacy of immune therapies and are associated with the development of resistance [36]. In this study, sequencing revealed that the expression of the immune checkpoint TIM-3 ligand, Galectin-9, was significantly upregulated in senescent fibroblasts. Galectin-9 is a ligand for the T cell surface immune checkpoint TIM-3, which plays a crucial role in T cell immune tolerance through the TIM-3/Galectin-9 signaling pathway, akin to the PD-1/PD-L1 pathway [37–39]. Furthermore, research has indicated that activation of the TGF-β/Smad3 signaling pathway significantly upregulates Galectin-9 expression [40, 41], thereby inducing the formation of a suppressive TME [24]. We found that fibroblasts in a tsRNA-induced senescent state not only exhibited a significant increase in Galectin-9 expression but also an increase in its secretion. Co-culture experiments confirmed that high Galectin-9 expression contributed to the suppression of CD8^+^ T cell function. Moreover, only by restoring FOXO3 expression and simultaneously inhibiting Galectin-9 could the cytotoxic function of CD8^+^ T cells be fully restored.

**Fig. 8:**
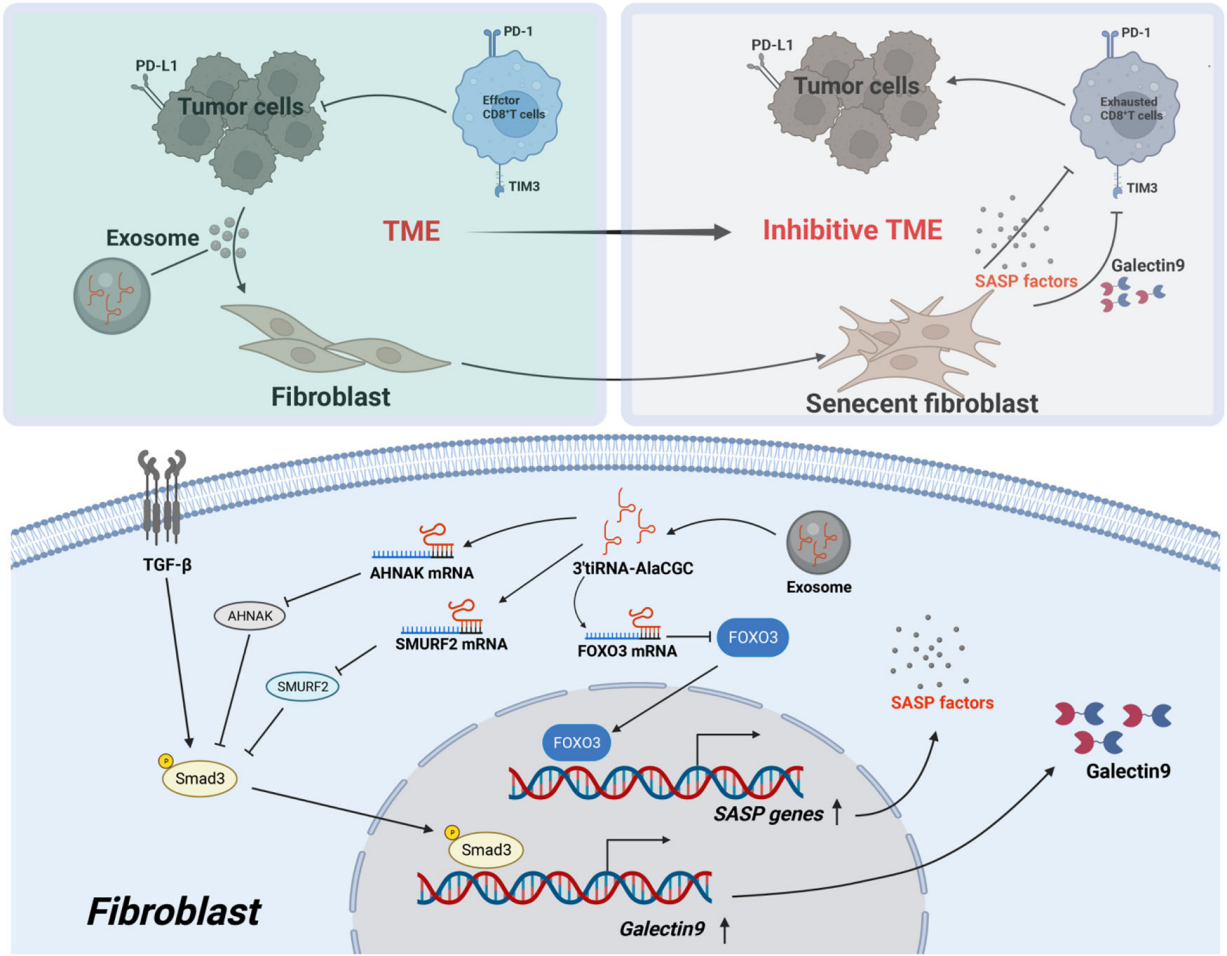
Diagram of the mechanism by which 3′ tiRNA-AlaCGC formation inhibits the tumor microenvironment.

In summary, based on clinical studies and utilizing specific small RNA microarray technology, we found that the expression of 3′tiRNA-AlaCGC is associated with resistance to immune therapy. Mechanistically, LUAD employs a novel mechanism of immune tolerance to counteract immune therapy, regulating fibroblast senescence and Galectin-9 secretion through the exosomal 3′tiRNA-AlaCGC (Fig. 8). However, our study still has limitations. The specific molecular mechanisms by which 3′tiRNA-AlaCGC induces fibroblast senescence leading to resistance to immune therapy, and whether it also plays other roles affecting the TME, remain to be explored. Whether targeting 3′tiRNA-AlaCGC can enhance the efficacy of tumor immune therapy and improve patient prognosis remains a direction for future research.

## Method

### Ethical statement

This study was approved by the Ethics Committee of Zhongshan Hospital, Fudan University (B2021-128), and performed following the principles of the Declaration of Helsinki.

### Cell culture

LUAD cell lines A549, H23, H1299, PC-9, H1975, normal lung cell line BEAS-2B, human embryonic kidney 293 (HEK293T) cells, and human lung fibroblast HFL1 were provided by the Chinese National Collection of Authenticated Cell Cultures (Shanghai, China). All cells were grown in high-glucose Dulbecco’s modified Eagle’s medium (KeyGEN, Nanjing, Jiangsu, China) containing 10% fetal bovine serum (Every Green, Huzhou, Zhejiang, China), 100 U/mL penicillin (Beyotime), and 0.1 mg/ml streptomycin (Beyotime).

### RNA extraction and quantitative real-time reverse transcription PCR

Total RNA was extracted from cells and tissues using the Trizol reagent (Life Technologies, USA). RNA Pretreatment Kit (#: AS-FS-005, Arraystar, USA) and rtStar™ First-Strand cDNA Synthesis Kit (#: AS-FS-003, Arraystar, USA) were used to build specific cDNA libraries for the quantification of tsRNA in cells and tissues. mRNA and 3′tiRNA-AlaCGC were reverse transcribed to cDNA using a PrimeScript RT Reagent Kit (Perfect Real-Time, Takara, Japan). Then, qPCR was performed with SYBR Premix Ex Taq (Takara). The expression levels of tsRNA was normalized to that of U6, and the expression levels of mRNAs were normalized to that of GAPDH. The primer used for detecting 3′tiRNA-AlaCGC was purchased from Aksomics (Shanghai, China), and the details of the other primers are shown In Supplementary Table 1.

### Cell proliferation, colony formation, migration, and invasion assays

For cell proliferation assay, the cells were seeded at a density of 1000 cells per well into 96-well plates and incubated for 0, 24, 48, 72, 96, and 120 h at 37°C, 5%CO_2_ condition. Cell viability was measured with CCK-8 (Beyotime) according to the manufacturer’s instructions.

For colony formation assay, the cells were seeded at a density of 500 cells in each well of a 6-well plate and then left to grow for 2 weeks, with media changes every 3-4 days until visible colonies were formed. Staining and Counting: Colonies are fixed with methanol, stained with crystal violet, and counted manually or using image analysis software.

For migration assay, Cells are grown to confluence in a 6-well plate. A “wound” was created by scratching the monolayer with a sterile pipette tip and then cells were allowed to migrate into the wound area for 48 hours. Migration is assessed by measuring the closure of the wound area using microscopy and image analysis software.

For invasion assay, cells were seeded into the upper chamber using serum-free medium, while the lower chamber was supplemented with serum-containing medium. Throughout 24 to 48 hours, cells migrate through the matrix barrier to the lower chamber. Those cells that successfully invade and reach the underside of the insert are subsequently fixed, stained, and quantified.

### Exosome isolation

Exosomes from the LUAD cell lines were extracted with ExoQuick exosome precipitation solution (SBI, USA) according to the manufacturer’s protocol. The extracted exosomes were validated through transmission electron microscopy and western blotting.

### Western blotting

Cells were lysed using RIPA buffer (Beyotime) supplemented with protease and phosphatase inhibitors (Beyotime). Protein concentrations were determined using an Enhanced BCA Protein Assay Kit (Beyotime), followed by denaturation in 5×SDS-PAGE loading buffer (EpiZyme, Shanghai, China) at 100 °C for 10 minutes. Proteins were then resolved by SDS-PAGE and transferred for western blot analysis using the specified antibodies. The antibodies used in the present study and their information are listed in the supplementary Table 2. The full and uncropped Western blot images are provided in Supplementary Fig. 2.

### Immunohistochemistry (IHC) and fluorescence in situ hybridization (FISH)

For IHC, CD8 antibody, and GTVisionTM III Detection System (GeneTech, Shanghai, China) were used for immunohistochemical staining. The intensity of positive staining was measured as described [42]. For in situ hybridization analysis, The probe and the FISH Kit were ordered from Boster Biological Technology (Wuhan, China). The expression of 3′tiRNA-AlaCGC was quantified using a visual grading system based on the degree of staining. The intensity of staining was divided into four grades: 0, negative; 1, weak; 2, moderate; and 3, strong. The positive cell percentages were classified as 0, negative; 1, 1 – ≥25%; 2, 25–50%; 3, 50–75%; and 4, >75%. A weighted staining score was calculated by multiplying the percentage of the positive cells and the grade of the staining intensity. Finally, all samples were assigned to two levels according to the score: < 3, low expression; ≥ 3, high expression.

### RNA immunoprecipitation (RIP) assay

RIP assay was performed using the Magna RIP RNA-Binding Protein Immunoprecipitation Kit (Millipore, Boston, USA) following the manufacturer’s protocol. In brief, cells were lysed in RIP lysis buffer and incubated with 20 μL of protein AGO2 antibody or IgG antibody (Abcam, ab172730, 1:100) at 4 °C overnight. RNA was purified and detected by qRT-PCR.

### RNA pull-down assay

Design and synthesize a biotin-labeled 3’tiRNA-AlaCGC probe using standard RNA synthesis methods, labeling the 3’ end with biotin (Genomeditech, Shanghai). After synthesis, purify the probe by HPLC or PAGE to ensure high purity and purified. Approximately 2 × 10^7^ cells were disintegrated in lysis buffer containing 80 U/mL RNasin Plus RNase Inhibitor (Promega). Cell extract was incubated for 4 h with biotinylated RNA probe and treated with streptavidin-coupled agarose beads (Invitrogen, Carlsbad, USA) for 2 h at 4 °C with gentle rotation. The beads are washed with low- and high-salt buffers to remove non-specific binding, and specifically bound RNA is extracted using TRIzol. Reverse transcription is performed to generate cDNA, followed by qPCR to quantify mRNA levels.

### Dual-luciferase reporter assay

We cloned the binding regions of FOXO3, AHNAK, and SMURF2 with 3′tiRNA-AlaCGC sequences, into the phy-811@7 dual luciferase reporter vector (Hanyin Technology, Shanghai, China). HEK293T cells were seeded on a polylysine-treated 24-well plate at 60–70% confluence. After 24 h, the cells were co-transfected with 200 nM 3′tiRNA-AlaCGC mimics and 400 ng of the wild-type or mutant plasmids constructed as above with Lipo8000 (Beyotime) as the transfection reagent. Forty-eight hours later, the cells were collected, and the dual-luciferase reporter assays were conducted with a Luciferase Reporter Gene Assay Kit (Beyotime). Lastly, luciferase activity was detected with a Microplate spectrophotometer (Bio-Rad, Hercules, CA, USA).

### Cytokine measurement

Cytokine production in the supernatants of HFL1 cells expressing different 3′tiRNA-AlaCGC was measured using the human cytokine/chemokine microarray (Luminex, USA). Additionally, the concentrations of interleukin-1β (IL-1β), IL-8, CSF2, and Galectin-9 in the HFL1 cell supernatants, as well as the concentrations of IFN-γ and GZMB secreted by CD8^+^ T cells under various co-culture conditions, were measured by enzyme-linked immunosorbent assay (ELISA). The ELISA kits used are listed in Supplementary Table 2.

### Co-culture of LUAD cells, CD8^+^T cells and HFL1 fibroblasts

A total of 1 × 10^6^ HFL1 cells were seeded in the lower chamber of six-well Transwell chambers (Corning, USA), and 5 × 10^5^ LUAD cells were seeded in the upper chamber. After 48 h, the supernatant and macrophages were collected for further analysis. After the isolation of CD8^+^ T cells from healthy donors by using anti-CD8 immunomagnetic beads, cells were stimulated with anti-CD3 (10 μg/mL), anti-CD28 (1.5 μg/mL), and IL-2 (200 U/mL) and cultured with supernatant from the co-culture system for 48 h. The cytotoxic capacity and exhaustion status of CD8^+^ T cells were assessed through ELISA and flow cytometry.

### Mice

Animal research was approved by the Animal Ethics Committee of Zhongshan Hospital and the experiments were conducted by the guidelines (B2023-350R). Five-week-old male C57BL/6 and BALB/c nude mice were purchased from SPF Biotechnology Co., Ltd. (Beijing, China). The mice were maintained in a specific pathogen-free (SPF) environment. Under the guidelines and approvals granted by our ethics committee, the maximum allowable tumor size and burden for this study were set at a length and width not exceeding 2 cm and a volume limit of 2000 mm³. Tumor growth in the mice was monitored every three days.

### Arraystar small RNA microarray and RNA sequencing

To detect the expression of tsRNAs in tumor tissues of LUAD patients with different treatment responses, Arraystar small RNA microarray experiments were performed by Aksomics (Shanghai, China). In addition, RNA-sequencing was carried out by OE Biotech (Shanghai, China) to identify the target genes regulated by 3’tiRNA-AlaCGC.Volcano and Heat map plots were used to illustrate the expression profiles of different tsRNAs and mRNAs. Kyoto Encyclopedia of Genes and Genomes (KEGG) and Gene ontology (GO) analyses were used to identify the senescence and checkpoint-related genes and explore potential candidates for 3′tiRNA-AlaCGC.

### Flow cytometry

Cells for flow cytometry analyses were obtained from human blood and isolated mouse tissue. Cells were suspended in 100 μl buffer (PBS, 1%BSA, 0.05%NaN3) and fluorochrome-conjugated antibodies were added together. After 30 min surface antibody incubation at 4 °C, cells were washed three times. Intracellular molecular staining was performed using the Foxp3-Transcription Factor Staining Buffer Set (Thermo Fisher, 00-5123-43), per the manufacturer’s protocol. For intracellular cytokine detection, cells were stimulated in vitro with cell stimulation cocktail (phorbol myristate acetate, ionomycin, monensin and brefeldin A; Thermo Fisher) for 5 h. Cell were then fixed, permeabilized, and stained with anti-cytokine monoclonal antibodies. Fluorescence was acquired using a BD FACSCelesta Multicolor Flow Cytometer, and data were analyzed using FlowJo software.

### Senescence β-Galactosidase Staining

Senescence β-Galactosidase ((SA-β-gal) Staining staining was performed using the Senescence β-Galactosidase Staining Kit (Beyotime, China) according to the manufacturer’s protocol. In brief, the cells under different treatments were washed once with PBS, fixed, and stained by the reagents provided by the kit.

### RNA interference and plasmids

Plasmid for FOXO3 and SMURF2 overexpression were acquired from Genomeditech (Shanghai, China). The plasmid was utilized to subsequently infect HFL1 cells depending on the manufacturer’s directions. Cell lysates and total RNA were obtained 72 h post-transfection or infection, and western blotting analysis was employed to confirm the efficiency of AHNAK overexpression.

### Statistics and reproducibility

Statistical evaluations were performed using GraphPad Prism (version 8.0) and R software packages. Continuous variables between the two groups were compared using Student’s unpaired *t*-tests and Wilcoxon rank-sum tests. The association between 3′tiRNA-AlaCGC and CD8^+^T cells was assessed using Spearman’s rank correlation tests. The Kaplan–Meier method was employed for survival analysis, and differences were tested with the log-rank method. Independent predictors of prognosis were identified using a Cox proportional hazards model. Data are shown as mean values, with standard deviation indicated by error bars, unless specified otherwise. Significance was determined using two-tailed p values, with thresholds set at *p < 0.05, **p < 0.01, ***p < 0.001, and ns indicating non-significance. The experiments were conducted in triplicate.

## Supplementary information

supplementary Figure -1

Supplemtary Figure 2

Supplementary Table 1

Supplementary Table 2

Supplementary Table 3

## Data Availability

The datasets used and/or analysed during the current study are available from the corresponding author on reasonable request.
