# Correction: Tumor-derived exosomal tsRNA 3′tiRNA-AlaCGC in promoting fibroblast senescence and Galectin-9 secretion to induce immune tolerance in lung adenocarcinoma

**DOI:** 10.1038/s41420-025-02879-x

**Published:** 2026-01-13

**Authors:** Guangyin Zhao, Yuchen Zhang, Hongyu Zhang, Yifan Guo, Chang Xu, Di Ge, Jie Gu

**Affiliations:** 1 Department of Thoracic Surgery, Shanghai Geriatric Medical Center, 2560 Chunshen Road, Shanghai, China; 2 Department of Thoracic Surgery, Zhongshan Hospital, Fudan University, Shanghai, China

**Keywords:** Tumour immunology, Senescence

Correction to: *Cell Death Discovery* 10.1038/s41420-025-02695-3, published online 25 August 2025

During figure assembly, the image used in Figure 2 (panel D) was inadvertently misplaced due to a copy–paste error. We have now provided the corrected version of Figure 2. This correction does not affect the results or the overall conclusions of the article.

Incorrect Figure 2
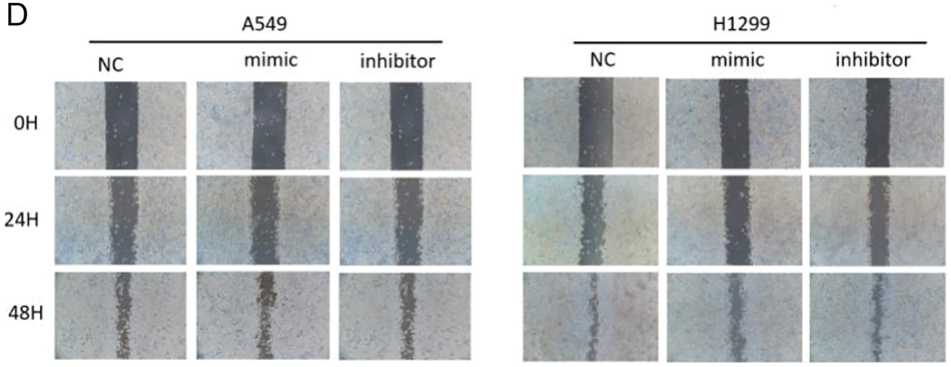


Correct Figure 2
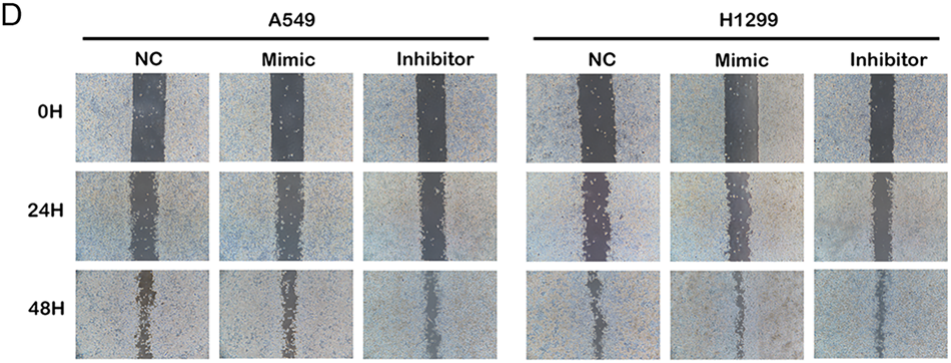


The original article has been corrected.

