# Supplementary figures and images for: Tumor-derived exosomal tsRNA 3′tiRNA-AlaCGC in promoting fibroblast senescence and Galectin-9 secretion to induce immune tolerance in lung adenocarcinoma
Source: Cell Death Discov. 2025 Aug 25;11:403. doi: 10.1038/s41420-025-02695-3 (PMC12379295; doi:10.1038/s41420-025-02695-3)

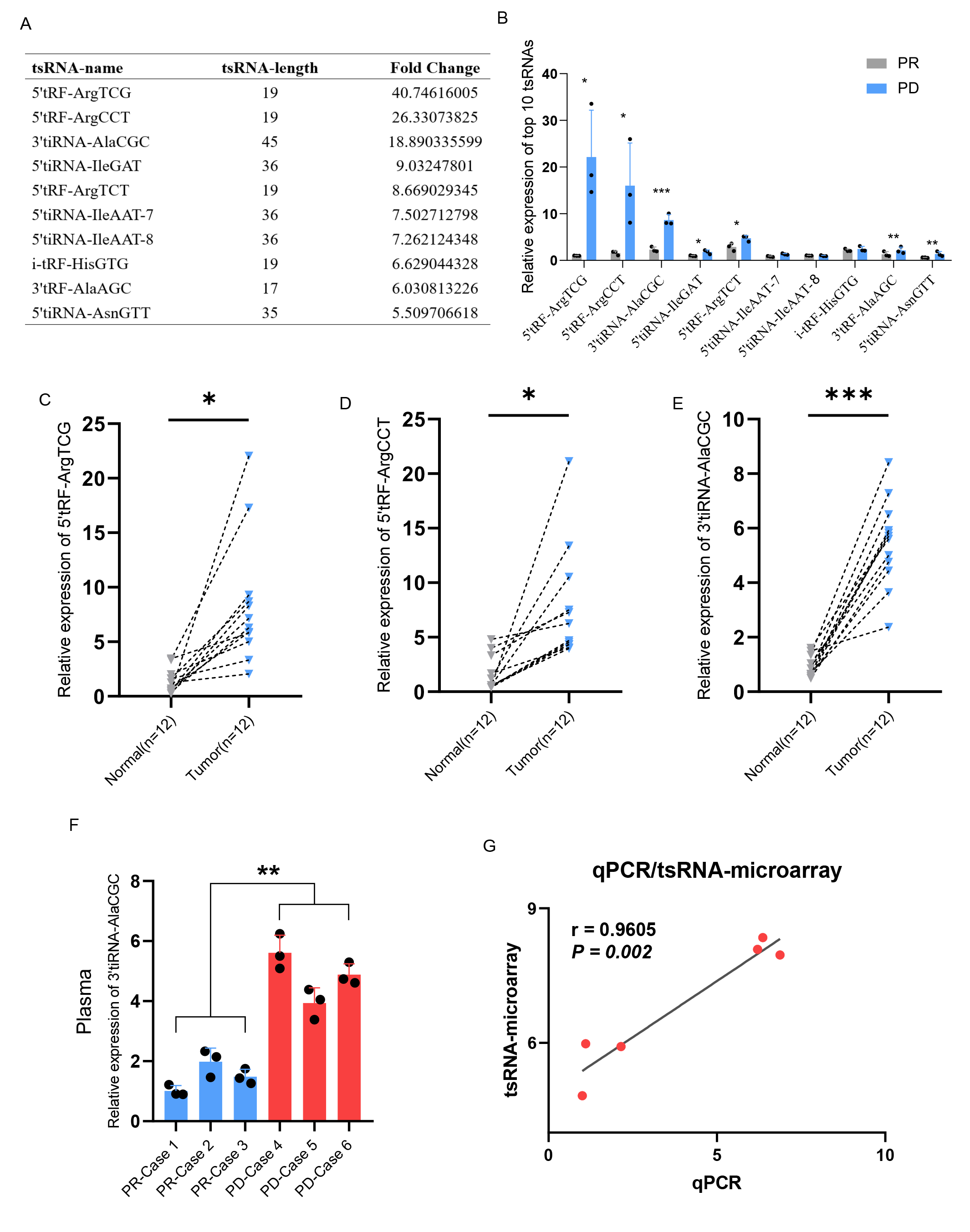

Supplement: Supplementary file 1 — supplementary Figure -1 [file 41420_2025_2695_MOESM1_ESM.tif]

Fig 3B

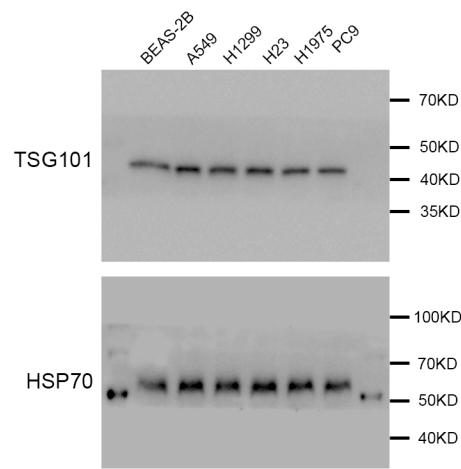

Fig 4H

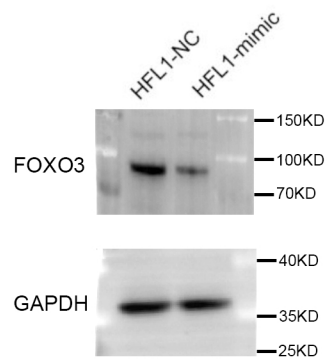

Fig 4N

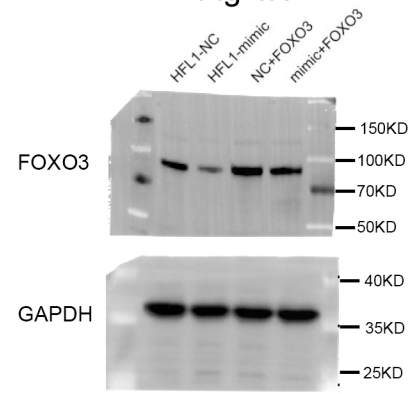

Fig 5D

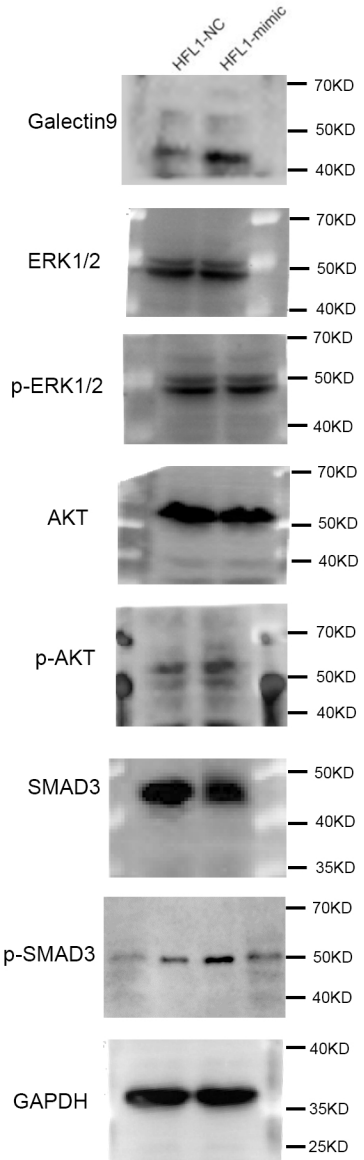

Fig 5E

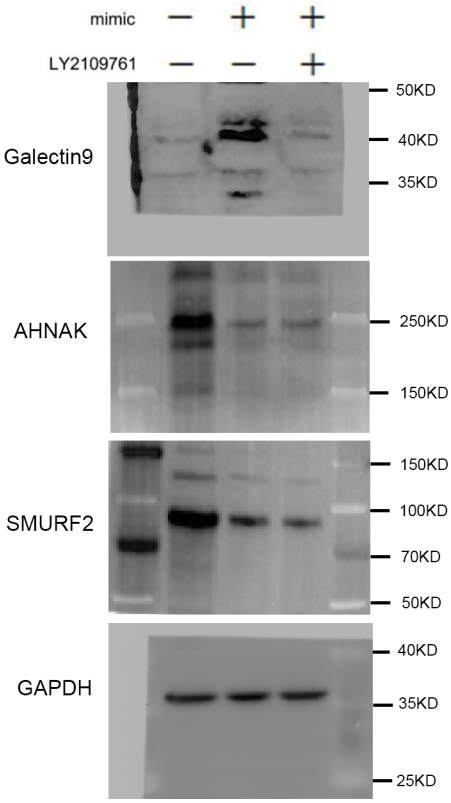

Fig 5F

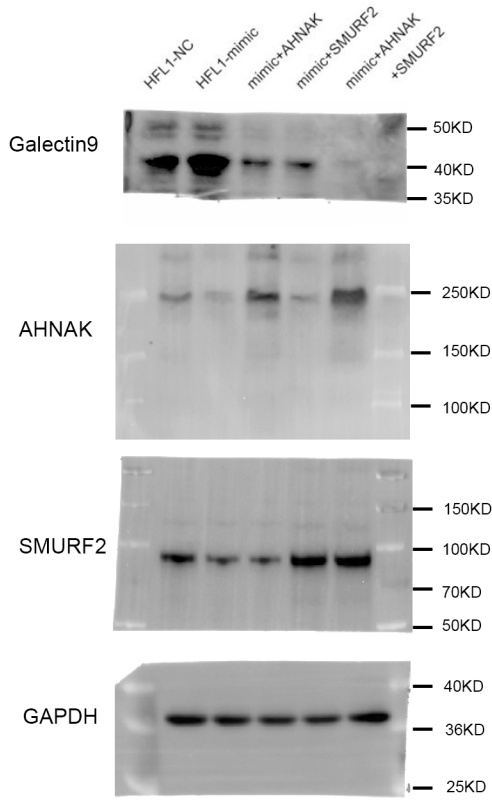

Supplement: Supplementary file 2 — Supplemtary Figure 2 [file 41420_2025_2695_MOESM2_ESM.pdf]
