# Supplementary material for: Tumor-derived exosomal tsRNA 3′tiRNA-AlaCGC in promoting fibroblast senescence and Galectin-9 secretion to induce immune tolerance in lung adenocarcinoma
Source: Cell Death Discov. 2025 Aug 25;11:403. doi: 10.1038/s41420-025-02695-3 (PMC12379295; doi:10.1038/s41420-025-02695-3)
Supplement: Supplementary file 3 — Supplementary Table 1 [file 41420_2025_2695_MOESM3_ESM.docx]

**Supplementary Table 1. Sequences of Primers for Quantitative real-time-Polymerase Chain Reaction Analysis**

| **Gene** | **Forward Primer: 5'-3'** | **Reverse Primer: 5'-3'** |
| --- | --- | --- |
| 3'tiRNA-AlaCGC | ACGATCTTCGCATGTACGAGG | CGATCTTGGTGGAGGAGCC |
| FOXO3 | AGAGATCAAAGAGTTCGCCGC | TCTTCATCCACTTCCACAGCG |
| GAPDH | TCGGAGTCAACGGATTTGGT | TTCCCGTTCTCAGCCTTGAC |
| U6 | GCTTCGGCAGCACATATACTAAAAT | CGCTTCACGAATTTGCGTGTCAT |
| 5'tRF-ArgTCG | GAGTGACCGCGTGGCCT | AGTGCAGGGTCCGAGGTATT |
| 5'tRF-ArgCCT | GAGTGCCCCAGTGGCCT | AGTGCAGGGTCCGAGGTATT |
| 5'tRF-ArgTCT | GAGTGGCTCCGTGGCGC | AGTGCAGGGTCCGAGGTATT |
| 5'tiRNA-IleGAT | GCTCAGTTGGTAAGAGCGTGG | AGTGCAGGGTCCGAGGTATT |
| 5'tiRNA-IleAAT-7 | CTCAGTTGGTCAGAGCGTGG | AGTGCAGGGTCCGAGGTATT |
| 5'tiRNA-IleAAT-8 | TCAGTCGGCTAGAGCGTGG | AGTGCAGGGTCCGAGGTATT |
| i-tRF-HisGTG | TCGGCAGGTGGTTAGTACTCT | AGTGCAGGGTCCGAGGTATT |
| 3'tRF-AlaAGC | GCCGAGCCCCAGTACCT | AGTGCAGGGTCCGAGGTATT |
| 5'tiRNA-AsnGTT | GCAATCGGTTAGCGCGTT | AGTGCAGGGTCCGAGGTATT |
