# Supplementary material for: Tumor-derived exosomal tsRNA 3′tiRNA-AlaCGC in promoting fibroblast senescence and Galectin-9 secretion to induce immune tolerance in lung adenocarcinoma
Source: Cell Death Discov. 2025 Aug 25;11:403. doi: 10.1038/s41420-025-02695-3 (PMC12379295; doi:10.1038/s41420-025-02695-3)
Supplement: Supplementary file 4 — Supplementary Table 2 [file 41420_2025_2695_MOESM4_ESM.docx]

**Supplementary Table 2.** **Details of antibodies and reagents used in this research**

| **Antibodies，ELISA kits and drugs** | **Company** | **Cat No** |
| --- | --- | --- |
| Anti- FOXO3 | Proteintech | 10849-1-AP |
| Anti-Galectin-9 | Abcam | ab69630 |
| SMAD3 | Proteintech | 66516-1-Ig |
| p-SMAD3 | Abcam | ab52903 |
| AKT | Proteintech | 60203-2-Ig |
| p-AKT | Proteintech | 80455-1-RR |
| ERK1/2 | CST | CST.4695s |
| p-ERK1/2 | CST | CST.4370s |
| Anti-AHNAK | Abcam | ab68556 |
| Anti-SMURF2 | Abcam | ab313470 |
| Anti-TSG101 | Abcam | ab125011 |
| Anti-HSP70 | Abcam | ab181606 |
| Anti-GAPDH | Abcam | ab8245 |
| FITC anti-human CD45 | Biolegend | 368507 |
| PE anti-human CD45 | Biolegend | 304007 |
| PE/Cyanine7 anti-human CD3 | Biolegend | 344815 |
| FITC anti-human CD4 | Biolegend | 317408 |
| APC anti-human CD8 | Biolegend | 344721 |
| APC/Cyanine7 anti-human CD25 | Biolegend | 302613 |
| Alexa Fluor® 647 anti-mouse/rat/human FOXP3 | Biolegend | 320014 |
| Brilliant Violet 421™ anti-human CD19 | Biolegend | 302234 |
| Hu CD3 BV421 | BD Pharmingen | 562427 |
| Anti-Human CD56 Antibody, Clone HCD56 | STEMCELL | 60021PE.1 |
| APC/Cyanine7 anti-human CD68 | Biolegend | 333822 |
| PE/Cyanine7 anti-human CD14 | Biolegend | 301814 |
| APC/Cyanine7 anti-human CD11c | Biolegend | 337217 |
| APC anti-mouse CD107a (LAMP-1) | Biolegend | 121613 |
| PE anti-mouse IFN-γ | Biolegend | 505807 |
| PE/Cyanine7 anti-mouse CD8a | Biolegend | 100722 |
| Brilliant Violet 421™ anti-mouse CD45 | Biolegend | 103133 |
| CD223 (LAG-3) Monoclonal Antibody (eBioC9B7W (C9B7W)), PE, eBioscience™ | Invitrogen | 12-2231-81 |
| APC anti-mouse CD279 (PD-1) | Biolegend | 109111 |
| Alexa Fluor® 647 anti-human/mouse Granzyme B | Biolegend | 515405 |
| Human Galectin-9 DuoSet ELISA | R&D system | DY2045 |
| Human GM-CSF DuoSet ELISA | R&D system | DY215 |
| Human IL-1 beta/IL-1F2 QuicKit ELISA | R&D system | QK201 |
| Human IL-8/CXCL8 Quantikine ELISA Kit | R&D system | D8000C |
| LY2109761 | Yeasen | 52605ES08 |
| Murine Anti-mPD-L1 mAb | InvivoGen | mpdl1c2-mab15-1 |
