# Supplementary material for: Tumor-derived exosomal tsRNA 3′tiRNA-AlaCGC in promoting fibroblast senescence and Galectin-9 secretion to induce immune tolerance in lung adenocarcinoma
Source: Cell Death Discov. 2025 Aug 25;11:403. doi: 10.1038/s41420-025-02695-3 (PMC12379295; doi:10.1038/s41420-025-02695-3)
Supplement: Supplementary file 5 — Supplementary Table 3 [file 41420_2025_2695_MOESM5_ESM.docx]

**Supplementary Table 3. Sequences of mimic and inhibitor**

|  | **Sequence 5'-3'** |
| --- | --- |
| Mimic | UUCGCAUGUACGAGGCCCCGGGUUCGACCCCCGGCUCCUCCACCA |
| Inhibitor | UGGUGGAGGAGCCGGGGGUCGAACCCGGGGCCUCGUACAUGCGAA |
